# Seasonal distribution of anisakid nematodes in fishes from Portuguese waters: Epidemiological update and implications to consumer risk

**DOI:** 10.1016/j.crpvbd.2026.100397

**Published:** 2026-06-09

**Authors:** Beatriz Mendes, Duarte Marques, Monalisa Medeiros, Leonor Abegão, Inês Mira, Ana Maria Munhoz, Sónia Ramos, Tiago Leandro Gomes

**Affiliations:** a Research in Veterinary Medicine (I-MVET), Faculty of Veterinary Medicine, Lusófona University, Lisbon University Centre, Campo Grande, 376, Lisbon, 1749-024, Portugal; b School of Psychology and Life Sciences, Lusófona University, Lisbon University Centre, Campo Grande, 376, Lisboa, 1749-024, Portugal; c MED-Mediterranean Institute for Agriculture, Environment and Development, University of Évora, Polo da Mitra, N^a^ Sr^a^ da Tourega, Évora, 7000-083, Portugal; d Veterinary and Animal Research Centre (CECAV), Faculty of Veterinary Medicine, Lusófona University, Campo Grande, 376, Lisbon, 1749-024, Portugal

**Keywords:** *Anisakis*, Marine fishes, Portugal, Seasonal variation, Distribution, PCR-RFLP, Food safety

## Abstract

The consumption of raw or undercooked fish has increased concerns regarding zoonotic parasites, particularly nematode larvae of the genus *Anisakis*. This study investigates the epidemiology of anisakid infections in commercially important fishes from Portuguese coastal waters, focusing on spatial and seasonal patterns. A total of 538 specimens of five fish species (*Trachurus trachurus*, *Scomber colias*, *Scomber scombrus*, *Merluccius merluccius*, and *Sardina pilchardus*) were collected from six localities during winter and summer 2024. Parasites were recovered from viscera and muscles by pepsin digestion, identified using polymerase chain reaction-restriction fragment length polymorphism (PCR-RFLP) targeting the internal transcribed spacer (ITS) region, and confirmed by sequencing. Generalized linear models were applied for data analysis. All species were infected with *Anisakis* spp. larvae, with significant differences among host species, host size, seasons, and regions. Most larvae were in the viscera (90.5–100%), with a smaller proportion in muscles. *Trachurus* showed the highest infection levels (45–100% prevalence; mean intensity up to 161 larvae per infected host). Molecular analysis of 424 larvae identified *Anisakis pegreffii* (56.6%) as the dominant species, followed by *A. simplex* (*sensu stricto*) (30.9%), putative hybrids (12.3%), and a single *Skrjabinisakis physeteris* larva. A significant north-south and seasonal gradient was observed, with *A. simplex* (*s.s.*) predominating in northern and winter samples, particularly in the muscles. These results suggest geographical and seasonal variations in *Anisakis* spp. distribution. Despite potential sampling bias, the presence of zoonotic species in fish muscles highlights the need for strict post-capture handling, freezing, and cooking practices to reduce consumer risk.

## Highlights

• Seasonal epidemiology of *Anisakis* spp. in 5 fish species from 6 localities off Portugal.
• *Anisakis pegreffii*, *A. simplex* (*s.s.*), hybrids, and *Skrjabinisakis physeteris* were identified molecularly.
• *Anisakis simplex* (*s.s.*) predominated in northern localities during winter.
• *Anisakis simplex* (*s.s.*) frequently occurred in fish muscles, whereas *A. pegreffii* was mostly confined to viscera.

## Graphical abstract

**Figure:**
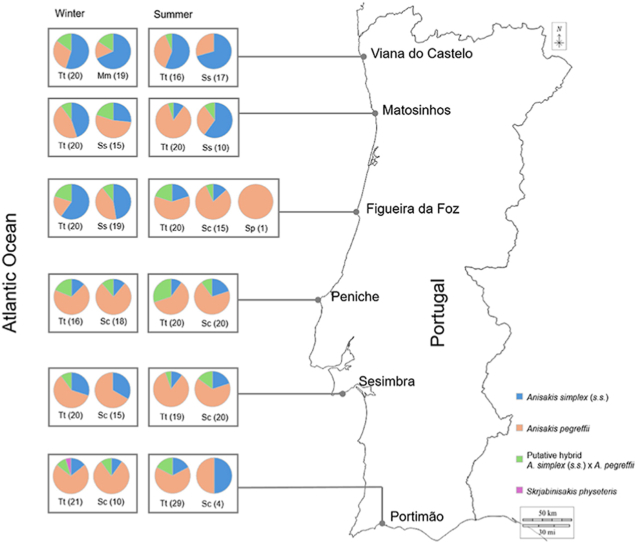

## 1 Introduction

Fishes and seafood are increasingly recognized by consumers as an essential component of a healthy and balanced diet, contributing approximately 17% of global animal-derived protein (Murthy et al., 2023; Nonković et al., 2025), and public health strategies have actively encouraged greater fish consumption (Murthy et al., 2023). But these products may represent a risk to consumers as they can contain zoonotic fish-borne agents, such as the larval parasites of the genus *Anisakis*, which are recognized by the European Food Safety Authority (EFSA) as a major parasitological zoonotic risk associated with fish consumption and are ranked among the top ten food-borne parasites (EFSA Panel on Biological Hazards (BIOHAZ), 2010, EFSA Panel on Biological Hazards (BIOHAZ), 2024; Nonković et al., 2025).

*Anisakis* spp. are marine parasitic nematodes with a complex life cycle involving multiple hosts. Adults live in the stomachs of cetacean definitive hosts, releasing eggs through the host’s faces that hatch into free-swimming larvae when they reach the water column. These are then ingested by micro crustacean intermediate hosts and subsequently accumulate in the muscles and viscera of fish and cephalopods, which act as paratenic hosts (Gomes et al., 2020; Audicana, 2022; Nonković et al., 2025). These nematodes are widely distributed worldwide and can cause serious pathological conditions in humans (Mattiucci et al., 2018; Nonković et al., 2025). Humans become accidental hosts of *Anisakis* spp. by ingesting third-stage larvae (L3) in raw or lightly processed seafood. Although the larvae cannot develop in humans, they can cause anisakiasis, a condition mainly characterized by gastrointestinal symptoms and, in some cases, allergic reactions (Baptista-Fernandes et al., 2017; Audicana, 2022; Nonković et al., 2025). The most reported species are *A. simplex* (*sensu stricto*), distributed in the northern hemisphere, especially in the North Atlantic and Pacific, and *A. pegreffii*, in the Mediterranean Sea and the Sea of Japan (Mattiucci et al., 2008, 2018; Gomes et al., 2020). A possible temperature-related distribution pattern of these two sibling species has been suggested in Japan (Gomes et al., 2023a, 2023b).

Anisakiasis is an emerging zoonosis, with 90% of reported cases occurring in Japan, where raw fish dishes such as *sushi* and *sashimi* are traditionally consumed. In Europe, most cases are documented in Spain and Italy, where marinated fish preparations are common, while fewer cases have been reported in countries like the Netherlands and Germany (EFSA Panel on Biological Hazards (BIOHAZ), 2010; Audicana, 2022). In Spain, the detection of allergic reactions to *Anisakis* spp. indicates ongoing exposure and has contributed to an increasing avoidance of fish among consumers concerned about the presence of larvae in fresh products (Bao et al., 2018; Asatryan et al., 2023).

In Portugal, a fish-based diet is deeply rooted in history and culture, and the apparent *per capita* consumption of fishery products stands out as the highest in the European Union, with approximately 55 kg consumed per year (EUMOFA, 2024). Traditional Portuguese eating habits include the consumption of whole, ungutted grilled fish (such as sardines, *Sardina pilchardus*, and small Atlantic horse mackerels, *Trachurus trachurus*), as well as boiled or stewed visceral organs like roe and liver, which are usually associated with a higher presence of anisakid larvae (Gomes et al., 2020). Despite not being a traditional part of the Portuguese diet, raw fish specialties have recently been introduced. A survey conducted in Porto (northern Portugal) involving individuals who consumed raw fish (*n* = 421) assessed their sociodemographic and health characteristics, and estimated that raw fish accounted for approximately 10% of all fish consumed, with an annual intake of 6.3 kg per person (Golden et al., 2022). This new reality, along with the globalization of travel and trade (Eiras et al., 2018), has increased the risk of this previously irrelevant zoonotic disease.

Surprisingly, Portugal is considered a low-prevalence country as far as allergy to *Anisakis* spp. is concerned, but the increased exposure of the population to parasitized fish can be associated with an increase in the incidence of sensitization and allergy (Rama and Silva, 2022). In the last decade, three infection cases of human anisakiasis have been diagnosed in the country during gastroscopy and reported in the literature for the first time (Baptista-Fernandes et al., 2017; Carmo et al., 2017; Bernardo and Castro-Poças, 2018).

The true burden of fish anisakiasis remains largely underestimated or unknown in most countries, particularly in Portugal, primarily due to limitations in surveillance systems and underreporting. Published data in the country are often spatially and temporally limited, frequently focusing on single host species from a specific locality, with sampling conducted on a limited and non-systematic basis and often lacking a molecular approach. Concurrently, rapid changes in climatic factors may disrupt previously established host-parasite dynamics, particularly in aquatic ecosystems, leading to rapid shifts in parasite distributions, prevalences, and transmission patterns (Atroch et al., 2026). Additionally, recent transformations on Portuguese consumer dietary patterns may have changed the potential impact of anisakids on public health. Accordingly, the EFSA expert panel has emphasized the need for comprehensive epidemiological studies of fishes intended for human consumption to better characterize the associated zoonotic risk and support effective prevention and control strategies (EFSA Panel on Biological Hazards (BIOHAZ), 2010, 2024).

In this context, the main objective of this study was to generate comprehensive epidemiological data on anisakids in fishes commonly consumed in Portugal, across two seasons (summer and winter) and multiple localities, focusing on molecular identification of larvae. Based on these data, implications for potential health risk to the Portuguese population are discussed.

## 2 Materials and methods

### 2.1 Sampling and parasitological data examination

**Fig. 1:**
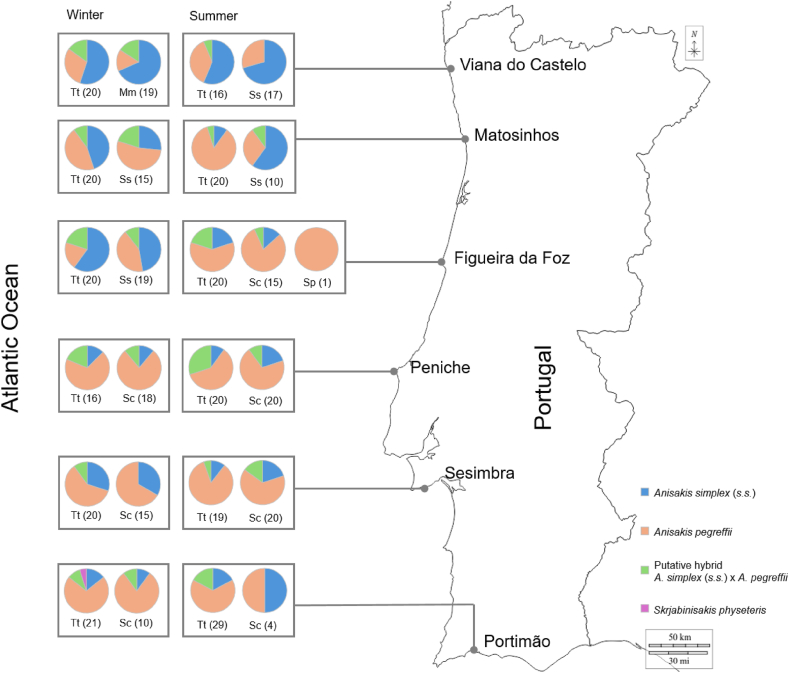
Geographical localities of sampling and respective anisakid species’ proportions. The number of larvae molecularly identified (PCR-RFLP) are expressed within parentheses next to the abbreviations of host names. *Abbreviations*: Mm, *Merluccius merluccius*; Sp, *Sardina pilchardus*; Sc, *Scomber colias*; Ss, *Scomber scombrus*; Tt, *Trachurus trachurus*.

Locally captured fishery products from six localities around Portugal (Fig. 1) were collected directly from first-sale establishments either immediately after landing or on the following day, during the winter (January to March) and summer (July and August) of 2024. Only one fish species (*Trachurus trachurus*) was sampled consistently across all localities and seasons, whereas other species were sampled opportunistically.

Fishes were always kept on ice and shipped to our laboratory, where they were immediately processed or, in some cases, frozen prior to later analysis. All fish specimens were individually measured (fork length) and weighed prior to dissection, during which the visceral organs (particularly digestive tract, liver, gonads and spleen) were removed, and the muscle tissue was filleted by separating the flesh from the spine of Atlantic horse mackerels (*Trachurus trachurus*), Atlantic chub mackerels (*Scomber colias*), Atlantic mackerels (*Scomber scombrus*) and Atlantic sardines (*Sardina pilchardus*). Atlantic hakes (*Merluccius merluccius*) were already eviscerated when obtained; therefore, only the muscles were examined. The viscera and muscles of each fish were then separately and individually subjected to pepsin digestion inside plastic containers (6 g pepsin 1:10,000 from porcine stomach mucosa, 7 ml HCl, 8 g NaCl, 1 L distilled water, incubated overnight at 38 °C in an incubator or water bath). The muscles (fish fillets) were previously sliced into smaller pieces, and all samples were occasionally stirred during incubation to facilitate digestion. All tissues were considered sufficiently digested when converted into free fibres or fragments no larger than a few millimetres. Anisakid larvae were then detected, collected, counted and fixed in 70% ethanol for further molecular analysis and identification of some specimens to the species level.

The ecological terminology used, such as locality (geographical locale) and site of infection (location in the host), as well as quantitative descriptors of parasite populations, such as prevalence (percentage of infected hosts) and mean intensity (total number of larvae found divided by the number of infected hosts), were defined according to Bush et al. (1997). The relative intensity reflects the mean fraction (%) of larvae detected in a specific site of infection, while the standard deviation was used to express the variability of the estimates.

### 2.2 Parasite identification

Polymerase Chain Reaction - Restriction Fragment Length Polymorphism (PCR-RFLP) was conducted for the molecular identification of larvae to the species level. In general, given the large number of samples available, only a subset of randomly selected, macroscopically indistinguishable larvae (*Anisakis* Type I *sensu* Berland, 1961 larvae) were analysed from each pool, which was represented by the anisakids collected from each site of infection (viscera and muscles), per sampling locality and season for each host species. For this selection procedure, the Sampling option of Microsoft Excel Data Analysis was used. In some cases, when fewer than 10 larvae were collected from each pool, all of them were molecularly identified. In addition, one larva morphologically distinct (*Anisakis* Type II *sensu* Berland, 1961 larvae) was also selected for molecular identification.

#### 2.2.1 DNA extraction and PCR

Genomic DNA was extracted using NZY Tissue gDNA Isolation kit from NZYtech (Lisbon, Portugal) (protocol for animal tissues). The ITS region (ITS1-5.8S-ITS2) was amplified with primers A (5′-GTC GAA TTC GTA GGT GAA CCT GCG GAA GGA TCA-3′) and B (5′-GCC GGA TCC GAA TCC TGG TTA GTT TCT TTT CCT-3′) (Gomes et al., 2020). Generally, 1 μl of DNA sample was mixed with 0.625 μl of forward (10 μM) and reverse primer (10 μM), 10.25 μl of distilled water (DW), and 12.5 μl of Taq mix (NZYTaq II 2× Green Master Mix) to a total volume of 25 μl per reaction. Occasionally, 1 μl of forward and reverse primer (10 μM each), 9.5 μl of DW and 12.5 μl of Taq mix (NZYTaq II 2× Green Master Mix) were used in some samples (particularly summer samples) to improve amplification efficiency. Amplification was conducted using a qTOWER3™ Real-Time PCR Thermal Cycler (Analytik Jena, Jena, Germany) and the conditions used consisted of a denaturation step of 3 min at 95 °C, followed by 30 cycles at 94 °C for 30 s, 59 °C for 30 s, 72 °C for 20 s, and then 7 min at 72 °C, according to manufacturer’s instructions.

#### 2.2.2 RFLP analysis and DNA sequencing

Following amplification of the ITS region, RFLP was carried out using Hinf I and Hha I FastDigest restriction enzymes (Thermo Scientific, Waltham, MA, USA), according to manufacturer’s recommendations. Hinf I enzyme was first used in all analysed samples, and in case the resulting band profile was compatible with *A. simplex* (*s.s.*) (2 bands of approximately 250 and 620 bp) or hybrid genotypes (*A. simplex* (*s.s.*) × *A. pegreffii*; 4 bands of 250, 300, 370 and 620 bp), those samples were further processed using Hha I, to rule out *A. berlandi*. Digested products were analysed by electrophoresis in a 1.5% agarose gel containing GelRed® Nucleic acid stain (Biotium, Fremont, CA, USA) and visualized by illumination with ultraviolet light. The resulting band pattern was analysed and compared with previously reported genetic keys (D’Amelio et al., 2000).

For a more robust species confirmation, the ITS region (ITS1-5.8S-ITS2) of some specimens was subsequently sequenced after purification using the previously cited primers by STAB Vida (Caparica, Portugal). The resulting nucleotide sequences were aligned and compared with sequences for anisakid species already available in GenBank, using the BLAST program (https://blast.ncbi.nlm.nih.gov/Blast.cgi). The newly generated nucleotide sequences were deposited and made available in GenBank under the accession numbers PX939965-PX939966, PX939976, PX940053, PX940055-PX940056, PX940097, PX940532 and PX940547.

### 2.3 Statistical analysis

Generalized linear models (GLM) were used to estimate both the probability of infection with anisakid larvae (prevalence) and infection intensity, either in the viscera or in the muscles, utilizing Jamovi software version 2.7 (https://www.jamovi.org) with the *GAMLj* module (https://gamlj.github.io/). In each case, host size (fork length in cm) was taken as a continuous covariate, while season, host species and locality were treated as factors. The model to analyse intensity included only infected fish. Because there was no data available regarding infection in the viscera of *M. merluccius*, this species was removed from this analysis. For prevalence, the binary response variable (presence or absence of anisakid larvae), was analysed using a binomial distribution with a logit link function. For intensity analyses, the number of larvae was initially modelled assuming a Poisson error distribution; however, due to high over-dispersion, a negative binomial error distribution was subsequently applied. *Post-hoc* comparisons among host species or localities were performed using Bonferroni-adjusted *P*-values.

Initial comparisons of parasite species frequencies between seasons and sampling localities were performed using Chi-square (*ꭓ*^2^) tests (or Fisher’s exact tests when expected frequencies were below 5) within the XLSTAT software (Addinsoft, New York, NY, USA). This analysis focused specifically on *A. simplex* (*s.s.*) and *A. pegreffii* from *T. trachurus*, as this was the only host species sampled across all localities and seasons (putative hybrids were excluded). To further validate and extend these findings while accounting for potential confounding factors, a binary logistic regression (generalized linear model, GLM) was applied on the full dataset of molecularly identified larvae. In this model, parasite species identity (*A. simplex* (*s.s.*) and *A. pegreffii*) was the dependent variable, host size (fork length in cm) was included as a continuous covariate, and locality, season, host species, and site of infection (viscera and muscles) were treated as fixed factors.

In all cases, the differences were considered statistically significant when the *P*-value was lower than 0.05.

## 3 Results

### 3.1 Parasitological data

Overall, 538 fishes of five species were analysed (Table 1), during two sampling periods (winter and summer), from six geographic localities (Fig. 1). *Trachurus trachurus* was the only host sampled across all localities and seasons, whereas *S*. *scombrus* and *S*. *colias* were primarily collected in the north and south of Portugal, respectively. Samples of *M*. *merluccius* and *S*. *pilchardus* were collected opportunistically from only one locality (and time of the year) each.

**Table 1:** Fish host sampling characteristics and total infection levels of anisakid larvae in *Trachurus trachurus*, *Scomber colias*, *Scomber scombrus*, *Merluccius merluccius*, and *Sardina pilchardus*.

Host	Locality	Season (month)	Days from capture to processing	*n*	Mean body weight (g)	Mean fork length (cm)	Prevalence (%)	Mean intensity
*T*. *trachurus*	Viana do Castelo	Winter (Jan)	3^a^	23	333 ± 66 (230–447)	29.8 ± 1.7 (26.5–32.5)	100	126.3 ± 62.5 (45–289)
		Summer (Jul)	2^a^	20	324 ± 52 (233–423)	30.3 ± 1.6 (26.5–32.5)	100	161.4 ± 62.9 (50–314)
	Matosinhos	Winter (Jan)	3	20	198 ± 28 (156–258)	26.1 ± 1.4 (24.0–29.0)	100	53.9 ± 29.4 (14–118)
		Summer (Jul)	2^a^	20	194 ± 28 (147–243)	25.4 ± 1.4 (230–280)	100	30.8 ± 35.9 (3–170)
	Figueira da Foz	Winter (Feb)	1	40	101 ± 118 (13–407)	182 ± 7.4 (10.5–33.0)	62.5	75.6 ± 144.6 (1–713)
		Summer (Jul)	1	20	276 ± 25 (235–326)	29.2 ± 1.1 (26.0–31.0)	100	142.6 ± 67.4 (56–349)
	Peniche	Winter (Mar)	1	25	127 ± 19 (86–164)	22.7 ± 1.2 (20.5–25.0)	96.0	10.9 ± 10.7 (1–42)
		Summer (Aug)	1	21	276 ± 41 (219–360)	27.8 ± 1.2 (26.0–30.5)	100	128.2 ± 80.2 (38–357)
	Sesimbra	Winter (Jan)	1	31	88 ± 55 (44–215)	18.9 ± 3.5 (15.0–25.0)	83.9	10.5 ± 8.6 (1–41)
		Summer (Jul)	1	20	219 ± 24 (182–283)	26.4 ± 0.9 (24.5–28.0)	100	40.3 ± 16.4 (16–84)
	Portimão	Winter (Jan)	1	20	205 ± 24 (162–237)	25.2 ± 1.2 (23.5–28.5)	100	25.9 ± 11.4 (4–55)
		Summer (Aug)	1	20	72 ± 14 (54–97)	18.2 ± 1.1 (16.5–20.5)	45.0	4.6 ± 5.2 (1–16)
*S*. *colias*	Figueira da Foz	Summer (Jul)	1	20	232 ± 26 (198–296)	27.5 ± 0.9 (25.0–29.0)	100	28.7 ± 25.6 (1–86)
	Peniche	Winter (Mar)	1	25	167 ± 33 (58–221)	25.5 ± 1.9 (18.5–28.5)	100	22.2 ± 16.6 (2–66)
		Summer (Aug)	1	20	382 ± 45 (311–463)	30.7 ± 0.8 (29.5–28.5)	100	82.2 ± 41.8 (23–166)
	Sesimbra	Winter (Jan)	1	21	263 ± 60 (164–378)	27.9 ± 1.9 (24.5–31.5)	100	24.4 ± 24.8 (2–93)
		Summer (Jul)	1	19	223 ± 44 (131–305)	25.8 ± 1.6 (22.0–28.5)	94.7	17.2 ± 9.9 (4–41)
	Portimão	Winter (Jan)	1	20	241 ± 35 (181–297)	27.0 ± 1.1 (25.0–28.5)	20.0	4.5 ± 2.1 (1–6)
		Summer (Aug)	1	20	69 ± 7 (53–85)	18.8 ± 0.7 (17.5–20.0)	15.0	1.3 ± 0.5 (1–2)
*S*. *scombrus*	Viana do Castelo	Summer (Jul)	2^a^	20	290 ± 59 (185–491)	31.8 ± 2.4 (26.5–37.0)	95.0	27.5 ± 13.2 (8–51)
	Matosinhos	Winter (Jan)	3	21	268 ± 39 (199–363)	29.3 ± 1.1 (27.0–31.5)	85.7	13.8 ± 13.9 (1–53)
		Summer (Jul)	2^a^	20	173 ± 21 (141–205)	25.4 ± 1.1 (22.0–26.5)	85.0	4.4 ± 3.7 (1–15)
	Figueira da Foz	Winter (Feb)	1	12	251 ± 52 (175–321)	28.7 ± 2.1 (26.0–31.5)	100.0	9.3 ± 5.3 (2–19)
*M*. *merluccius*	Viana do Castelo	Winter (Jan)	3^a^	20	397 ± 98 (240–673)	37.9 ± 3.0 (32.0–45.0)	65.0^b^	2.1 ± 1.0 (1–4)^b^
*S*. *pilchardus*	Figueira da Foz	Summer (Jul)	1	20	39 ± 5 (29–48)	14.8 ± 0.7 (13.5–16.0)	5.0	1.0 ± 0.0 (1–1)

*Note*: Mean values ± standard deviations are followed by the range in parentheses; *n* represents the number of specimens analysed.

a Samples were frozen (−18 °C) prior to later analysis. All other samples were analysed fresh.

b Numbers represent larvae found in muscles only, as viscera were not analysed.

All fish species were infected with *Anisakis* L3 larvae, although prevalences varied considerably. *Sardina pilchardus* had a relatively low prevalence (5%, corresponding to 1 infected fish out of 20 analysed) and only one larva was detected (in the viscera). The prevalence in *T*. *trachurus* varied between 45% and 100%, while mean intensities ranged from approximately 5 to 161 larvae per infected host. In contrast, prevalence in *S*. *colias* varied between 15% and 100%, and fish harboured approximately 1–82 larvae on average (Table 1). Collected larvae were mostly located in the viscera of the fish (representing a proportion of intensity of 90.5–100%) rather than in the muscles (0–9.5%) (Table 2). Prevalence specifically in the muscles was highest in *S*. *colias* (0–85%) and *T*. *trachurus* (0–75%), followed by *M*. *merluccius* (65%) and *S*. *scombrus* (14.3–41.7%). The mean intensity in the muscles of one of the groups of Atlantic chub mackerel (*S. colias*) reached approximately 6 larvae per infected host.

**Table 2:** Prevalence, mean intensity, and relative intensity levels of L3 anisakid larvae in the viscera and muscles of their fish hosts *Trachurus trachurus*, *Scomber colias*, *Scomber scombrus*, *Merluccius merluccius*, and *Sardina pilchardus*.

Host	Locality	Season	Viscera			Muscles		
			Prevalence (%)	Mean intensity	Relative intensity (%)	Prevalence (%)	Mean intensity	Relative intensity (%)
*T*. *trachurus*	Viana do Castelo	W (*n* = 23)	100	124.5 ± 61.5 (45–287)	98.6	60.9	2.9 ± 3.1 (1–12)	1.4
		S (*n* = 20)	100	161.3 ± 62.9 (49–312)	99.0	60.0	2.7 ± 2.0 (1–6)	1.0
	Matosinhos	W (*n* = 20)	100	52.6 ± 29.5 (12–115)	97.7	65.0	1.9 ± 1.1 (1–5)	2.3
		S (*n* = 20)	100	29.3 ± 32.8 (3–155)	95.3	35.0	4.1 ± 4.6 (1–15)	4.7
	Figueira da Foz	W (*n* = 40)	62.5	73.6 ± 138.3 (1–678)	97.3	30.0	4.3 ± 9.3 (1–35)	2.7
		S (*n* = 20)	100	141.0 ± 67.2 (56–346)	98.9	75.0	2.1 ± 0.8 (1–3)	1.1
	Peniche	W (*n* = 25)	96.0	10.7 ± 10.2 (1–39)	97.7	12.0	2.0 ± 0.8 (1–3)	0.3
		S (*n* = 21)	100	127.0 ± 79.8 (38–355)	99.0	66.7	1.9 ± 1.0 (1–4)	1.0
	Sesimbra	W (*n* = 31)	83.9	10.1 ± 8.2 (1–38)	96.0	16.1	1.8 ± 0.9 (1–3)	4.0
		S (*n* = 20)	100	39.8 ± 16.3 (15–84)	98.9	35.0	1.3 ± 0.5 (1–2)	1.1
	Portimão	W (*n* = 20)	100	25.4 ± 13.8 (4–55)	98.1	30.0	1.7 ± 1.1 (1–4)	1.9
		S (*n* = 20)	45.0	4.6 ± 5.2 (1–16)	100	0	0	0
*S*. *colias*	Figueira da Foz	S (*n* = 20)	100	28.4 ± 25.5 (1–86)	99.1	25.0	1.0 ± 0.0 (1–1)	0.9
	Peniche	W (*n* = 25)	100	21.9 ± 16.7 (2–66)	98.6	20.5	1.6 ± 0.5 (1–2)	1.4
		S (*n* = 20)	100	76.9 ± 41.8 (22–164)	93.6	85.0	6.2 ± 4.3 (1–16)	6.4
	Sesimbra	W (*n* = 21)	100	24.2 ± 24.8 (2–92)	99.0	19.1	1.3 ± 0.4 (1–2)	1.0
		S (*n* = 19)	94.7	16.3 ± 9.0 (3–35)	95.1	36.8	2.1 ± 1.7 (1–6)	4.9
	Portimão	W (*n* = 20)	20.0	4.5 ± 2.1 (1–6)	100	0	0	0
		S (*n* = 20)	15.0	1.3 ± 0.5 (1–2)	100	0	0	0
*S*. *scombrus*	Viana do Castelo	S (*n* = 20)	95.0	26.1 ± 12.5 (8–51)	94.6	40.0	3.5 ± 4.0 (1–14)	5.4
	Matosinhos	W (*n* = 21)	85.7	13.5 ± 13.8 (1–53)	98.0	14.3	1.7 ± 0.5 (1–2)	2.0
		S (*n* = 20)	85.0	3.9 ± 3.7 (1–15)	90.5	25.0	1.4 ± 0.5 (1–2)	9.5
	Figueira da Foz	W (*n* = 12)	100	8.6 ± 5.3 (2–18)	92.0	41.7	1.8 ± 5.3 (1–3)	8.0
*M*. *merluccius*	Viana do Castelo	W (*n* = 20)	NA	NA	NA	65.0	2.1 ± 1.0 (1–4)	NA
*S*. *pilchardus*	Figueira da Foz	S (*n* = 20)	5.0	1.0 ± 0.0 (1–1)	100	0	0	0

*Note*: Mean values ± standard deviations are followed by the range in parentheses.

*Abbreviations*: W, winter; S, summer; *n*, number of fish specimens analysed; NA, not applicable (in *M*. *merluccius*, the viscera were not analysed).

The GLM analysis (Supplementary Tables S1–S4) revealed that host size was the strongest predictor of parasite prevalence in the viscera (*ꭓ*^2^ = 102.2, *df* = 1, *P* < 0.001), with infection odds increasing significantly with fish length (OR = 1.66, 95% CI: 1.43–1.94). Additionally, host species (*ꭓ*^2^ = 68.2, *df* = 3, *P* < 0.001), locality (*ꭓ*^2^ = 76.2, *df* = 5, *P* < 0.001) and season (*ꭓ*^2^ = 12.0, *df* = 1, *P* < 0.001) also showed statistical significance to predict the presence or absence of anisakid larvae in the viscera. Regarding infection intensity, host species and locality also exerted significant effects on parasite counts (*ꭓ*^2^ = 292.4, *df* = 3, *P* < 0.001 and *ꭓ*^2^ = 28.3, *df* = 5, *P* < 0.001, respectively). Specifically, *T. trachurus* exhibited the highest parasite load, while *S. scombrus* showed an 89% lower expected parasite count (OR = 0.11, 95% CI: 0.08–0.14, *P* < 0.001).

**Fig. 2:**
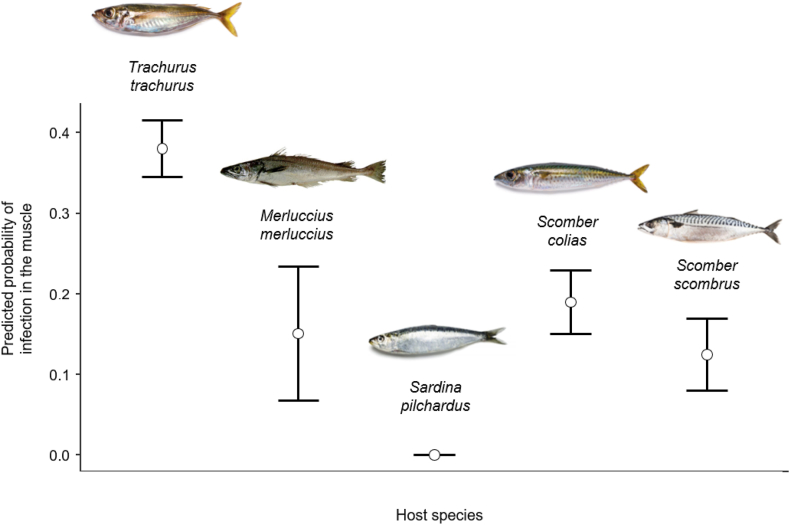
Predicted probability of the presence of anisakid larvae in the muscle tissue of various host species. Data points represent means ± standard error (SE) estimated from a binomial GLM (logit link function). Host fork length was included as a covariate in the model.

**Fig. 3:**
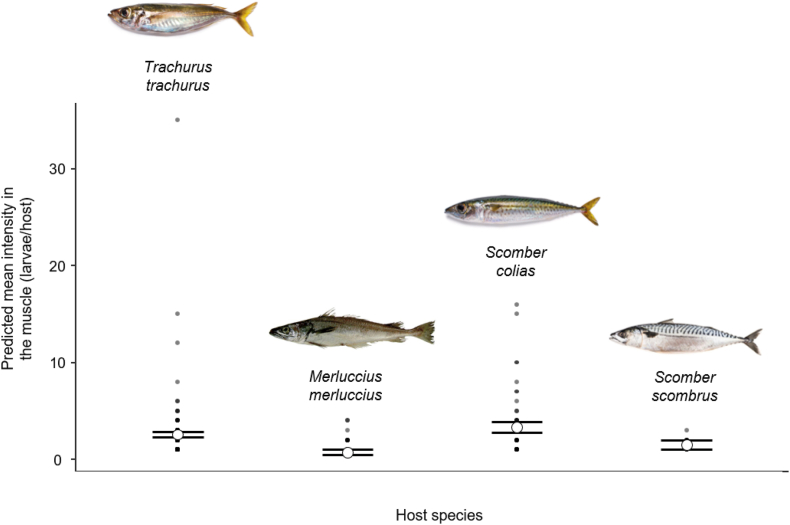
Estimated mean intensity of anisakid larvae infecting muscles of different host species. Results are expressed as the mean ± standard error (SE). Estimates were derived from a negative binomial GLM, with host fork length included as a covariate in the model.

Host size (*ꭓ*^2^ = 44.06, *df* = 1, *P* < 0.001), host species (*ꭓ*^2^ = 29.07, *df* = 4, *P* < 0.001) and locality (*ꭓ*^2^ = 16.54, *df* = 5, *P* = 0.005) were significant predictors of prevalence in the muscles, with *T. trachurus* showing the highest estimated infection prevalence (Fig. 2). Similarly, host size (*ꭓ*^2^ = 16.22, *df* = 1, *P* < 0.001) and host species (*ꭓ*^2^ = 16.57, *df* = 3, *P* < 0.001) were also significant factors for parasite intensity in the muscles (Fig. 3). Each unit increase in host length corresponded to an 11% increase in muscle parasite load (Incidence Rate Ratio = 1.11, 95% CI: 1.05–1.17). *Post-hoc* tests confirmed that anisakid intensity in the muscles of *T. trachurus* differed significantly from that of *M. merluccius* (*z* = 3.23, *P* = 0.007) as well as between this latter species and *S. colias* (*z* = −3.62, *P* = 0.002). No significant differences in infection intensity in the muscles were found between geographical sampling sites (*ꭓ*^2^ = 5.42, *df* = 5, *P* > 0.05).

### 3.2 Molecular identification

Overall, the analysed larvae were molecularly identified as *Anisakis pegreffii* (240/424, 56.6%), *A. simplex* (*s.s.*) (131/424, 30.9%), and putative hybrid genotypes (52/424, 12.3%) of these two sibling species (as reported by Gomes et al., 2020), as well as one larva (0.2%) identified as *Skrjabinisakis physeteris* (syn. *Anisakis physeteris*) (Table 3, Table 4, Fig. 1). Sequencing of the ITS regions (ITS1-5.8S-ITS2) from selected specimens of different RFLP band profiles confirmed the identity of the assigned species (Supplementary Table S5).

**Table 3:** Species of larvae identified by PCR-RFLP from samples of *Trachurus*, *Scomber colias*, *Scomber scombrus*, and *Merluccius*, collected during the winter.

Host	Locality (month)	Site in fish	*N*	*n*	Number of worms per species			
					*A*. *simplex* (*s.s.*)	*A. pegreffii*	Hybrid^a^	*S*. *physeteris*
*T*. *trachurus*	Viana do Castelo (Jan)	Viscera	2864	10	4	5	1	0
		Muscles	41	10	7	1	2	0
	Matosinhos (Jan)	Viscera	1052	10	3	7	0	0
		Muscles	25	10	6	2	2	0
	Figueira da Foz (Feb)	Viscera	1839	10	3	4	3	0
		Muscles	52	10	9	0	1	0
	Peniche (Mar)	Viscera	256	10	0	7	3	0
		Muscles	6	6	2	4	0	0
	Sesimbra (Jan)	Viscera	263	10	1	7	2	0
		Muscles	11	10	5	5	0	0
	Portimão (Jan)	Viscera	507	11	1	8	1	1
		Muscles	10	10	2	7	1	0
*S*. *colias*	Peniche (Mar)	Viscera	547	10	0	9	1	0
		Muscles	8	8	2	5	1	0
	Sesimbra (Jan)	Viscera	508	10	3	7	0	0
		Muscles	5	5	2	3	0	0
	Portimão (Jan)	Viscera	18	10	1	8	1	0
*S. scombrus*	Matosinhos (Jan)	Viscera	243	10	4	3	3	0
		Muscles	5	5	0	5	0	0
	Figueira da Foz (Feb)	Viscera	103	10	7	2	1	0
		Muscles	9	9	2	6	1	0
*M*. *merluccius*	Viana do Castelo (Jan)	Muscles	27	19	13	3	3	0

*Abbreviations*: *N*, number of larvae available; *n*, number of larvae analysed.

a Putative hybrids *Anisakis simplex* (*s.s*.) × *A. pegreffii*.

**Table 4:** Species of anisakid larvae identified by PCR-RFLP from samples of *Trachurus trachurus*, *Scomber colias*, *Scomber scombrus*, and *Sardina pilchardus*, collected during the summer.

Host	Locality (month)	Site in fish	*N*	*n*	Number of worms per species		
					*A*. *simplex* (*s.s.*)	*A. pegreffii*	Hybrid^a^
*T*. *trachurus*	Viana do Castelo (Jul)	Viscera	3225	8	3	4	1
		Muscles	32	8	6	2	0
	Matosinhos (Jul)	Viscera	586	10	2	7	1
		Muscles	26	10	0	10	0
	Figueira da Foz (Jul)	Viscera	2819	10	1	6	3
		Muscles	32	10	3	6	1
	Peniche (Aug)	Viscera	2666	10	0	6	4
		Muscles	26	10	2	6	2
	Sesimbra (Jul)	Viscera	796	10	1	8	1
		Muscles	9	9	1	8	0
	Portimão (Aug)	Viscera	41	29	5	19	5
*S*. *colias*	Figueira da Foz (Jul	Viscera	568	10	0	9	1
		Muscles	5	5	2	3	0
	Peniche (Aug)	Viscera	1538	10	0	9	1
		Muscles	105	10	4	5	1
	Sesimbra (Jul)	Viscera	294	10	2	7	1
		Muscles	15	10	2	6	2
	Portimão (Aug)	Viscera	4	4	2	2	0
*S. scombrus*	Viana do Castelo (Jul)	Viscera	495	10	6	4	0
		Muscles	28	7	6	1	0
	Matosinhos (Jul)	Viscera	67	7	4	3	0
		Muscles	7	3	2	0	1
*S*. *pilchardus*	Figueira da Foz (Jul)	Viscera	1	1	0	1	0

*Abbreviations*: *N*, number of larvae available; *n*, number of larvae analysed.

a Putative hybrids *Anisakis simplex* (*s.s*.) × *A. pegreffii*.

Overall, *A. pegreffii* was the most prevalent parasite species in most of the analysed hosts, localities and sampling seasons. Nevertheless, *A*. *simplex* (*s.s.*) was the most prevalent species in *T*. *trachurus* in the northern localities of Figueira da Foz (60%), Viana do Castelo (55%) and Matosinhos (45%, same as *A. pegreffii*) during the winter, although this supremacy was only observed in Viana do Castelo (56.3%) during the summer (Table 3, Table 4, Fig. 1). The overall presence of larvae of *A. simplex* (*s.s.*) and *A. pegreffii* off Portugal differed significantly between seasons (winter and summer) in *T*. *trachurus* (*ꭓ*^2^ = 9.72, *df* = 1, *P* = 0.002). In this fish host, the number of identified larvae of *A. simplex* (*s.s.*) was significantly lower during the summer than in the winter. This pattern was further observed in Figueira da Foz and Matosinhos (Fisher’s exact test, *P* = 0.006 and *P* = 0.040, respectively).

*Anisakis simplex* (*s.s.*) was also the most frequently identified parasite species (50.8%) in *S. scombrus* and *M*. *merluccius* (68.4%). In contrast, the only larva detected in *S. pilchardus* was molecularly identified as *A. pegreffii*.

**Fig. 4:**
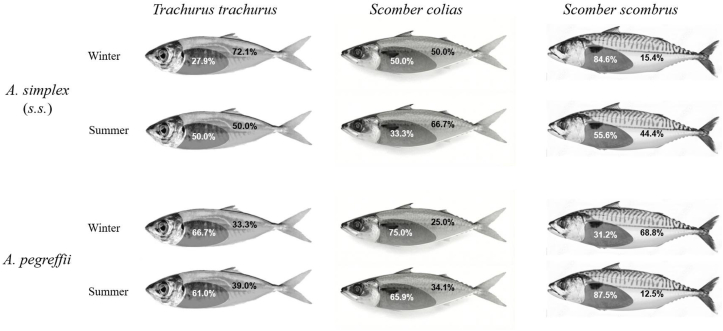
Relative abundance of *Anisakis* spp. larvae by anatomical location in *Trachurus trachurus*, *Scomber colias*, and *Scomber scombrus* collected from the sampling localities indicated in Fig. 1. Figures are based on the number of larvae located at a given site. Information on the viscera is represented with white figures on darkly shaded areas, and from the muscles with black figures on lightly shaded areas.

Regarding the anatomical distribution of the larvae, although *A. pegreffii* was generally the most prevalent sibling species both in the muscles and viscera of the fish, some exceptions are worth mentioning. In *T*. *trachurus*, during the winter off Viana do Castelo, Matosinhos, Figueira da Foz, and Sesimbra, *A. simplex* (*s.s.*) was the most frequently detected species in the muscles of the fish, and during the summer off Viana do Castelo. Larvae of *A. simplex* (*s.s.*) were generally more frequently detected in the muscles when compared to the viscera, and those of *A. pegreffii* were more frequent in the viscera than in the muscles, particularly in *T*. *trachurus* and *S. colias* (Fig. 4). The difference in the general prevalence of the two sibling species in the fish muscles between the two seasons was statistically significant in *T. trachurus* (ꭓ^2^ = 11.4, *df* = 1, *P* < 0.001).

Conversely, in *S*. *scombrus*, although *A. simplex* (*s.s.*) was the predominant species, the muscles of fish collected from off Matosinhos and Figueira da Foz during the winter harboured mainly *A. pegreffii* larvae.

Overall, *A*. *simplex* (*s.s.*) was more prevalent in *T*. *trachurus* collected in the north of the country (off Viana do Castelo, Matosinhos and Figueira da Foz) when compared with the southern localities (off Peniche, Sesimbra and Portimão) during the winter (*ꭓ*^2^ = 16.6, *df* = 1, *P* < 0.001), but not in the summer (*ꭓ*^2^ = 2.93, *df* = 1, *P* = 0.087).

A binary logistic regression model was fitted to the total pool of identified larvae (*n* = 370) and the model confirmed that parasite species identity was not significantly influenced by host size (*ꭓ*^2^ = 0.005, *df* = 1, *P* = 0.944) nor by host species (*ꭓ*^2^ = 3.850, *df* = 4, *P* = 0.427). Conversely, site of infection (*ꭓ*^2^ = 10.077, *df* = 1, *P* = 0.002), locality (*ꭓ*^2^ = 17.952, *df* = 5, *P* = 0.003) and season (*ꭓ*^2^ = 4.224, *df* = 1, *P* = 0.040) were statistically significant factors. For instance, all localities showed significantly lower odds of *A. simplex* (*s.s.*) occurrence compared to Viana do Castelo, particularly Peniche (*z* = −3.935, *P* < 0.001), followed by Sesimbra (*z* = −3.050, *P* = 0.002) and Portimão (*z* = −2.847, *P* = 0.004). Furthermore, the model indicated that the odds of larvae of *A. simplex* (*s.s.*) occurring in the viscera were significantly lower than in the muscles (*z* = −3.142, *P* = 0.002), regardless of host species or season (Supplementary Table S6).

Putative hybrid specimens (*A. simplex* (*s.s.*) × *A. pegreffii*) were also found in all localities and all hosts (except for *S*. *pilchardus*), both in the viscera and muscles, although generally in a small proportion (0–40%). A single larva of *Skrjabinisakis physeteris* was identified from the viscera of a specimen of *T*. *trachurus* collected from Portimão in winter.

## 4 Discussion

This study provides an updated and comprehensive assessment of the occurrence, distribution, and molecular characterization of *Anisakis* spp. in commercially important fish species from Portuguese coastal waters. The results suggest pronounced spatial and seasonal variations and confirm the co-occurrence of *A. simplex* (*s.s.*), *A. pegreffii*, and their putative hybrids. These findings highlight the ecological complexity of anisakid transmission in the transitional Atlantic region encompassing the waters surrounding the Iberian Peninsula and suggest the possible role of temperature in shaping the distributions of these two sibling species.

Sampling did not consistently follow a balanced design across species, localities, and seasons due to variability in the availability of landed fishery products across the country. Only *T*. *trachurus* was sampled systematically, whereas other species were collected opportunistically, in some cases restricted to a single locality or season (*S*. *pilchardus* and *M*. *merluccius*). All analysed fish species were infected with *Anisakis* L3 larvae, although prevalence and intensity varied considerably among host species (Table 1). In *T*. *trachurus*, prevalence was consistently close to 100%, except in specific localities during certain seasons (off Figueira da Foz, Sesimbra, and Portimão), where lower infection rates were likely associated with smaller host sizes. Mean intensities followed a similar pattern. These results are consistent with other surveys on this host in the region (Silva and Eiras, 2003; Lopes et al., 2020), although comparisons among samples may be limited by differences in fish size and weight.

Similarly, *S*. *colias* and *S. scombrus* exhibited relatively high infection levels, with prevalences generally close to 100%. Other studies have consistently reported high levels of parasitism in *Scomber* spp. off the Iberian Peninsula (Silva and Eiras, 2003; Levsen et al., 2017; Santos et al., 2017). Among our results, a notable exception was observed in *S. colias* collected off Portimão, which exhibited low prevalence in both summer (15%) and winter (20%). Fish sampled in this locality during summer were relatively small (mean fork length 18.8 cm), which would explain the low levels of parasitism, unlike those collected during winter (27.0 cm). Host size (and, consequently, age) plays a significant role in infection dynamics as demonstrated by generalized linear models in the present study. The lower infection levels observed in smaller fish possibly reflect reduced exposure time to infective stages and differences in diet and trophic position. In contrast, larger and older individuals, having experienced longer exposure periods and often occupying higher trophic levels, are more likely to accumulate parasitic larvae in their tissues over time (Bušelić et al., 2018; Levsen et al., 2018; Mattiucci et al., 2018).

In *M*. *merluccius*, only the muscles were analysed (with a prevalence 65%) because specimens of this species are usually eviscerated onboard immediately after capture to avoid spoilage, thus limiting direct comparisons with other species. A previous survey along the Portuguese coast reported a 100% overall prevalence in this host (Silva and Eiras, 2003), whereas in another, two sampling groups showed prevalences of 76.7% and 95.6%, respectively (Santos et al., 2022), although both viscera and muscles were surveyed. This suggests that general infection levels may potentially have been higher if data from viscera were included.

Although *S*. *pilchardus* exhibited a very low infection rate (5%), with only a single larva found in the viscera of one host, other studies in Portugal and northern Spain have reported higher infection rates in this host. A study in northern Portugal reported a prevalence of 21.8% and a mean intensity of 0.63 larvae per infected sardine (Silva and Eiras, 2003). Another work conducted off northwestern Spain and Portugal (ICES IXa) detected a prevalence of 62% in the viscera and 17% in the muscles of this host, but with a low mean intensity (1–2 larvae per infected host) (Levsen et al., 2018). These relatively low figures, particularly the intensities, are consistent with a mostly planktivorous diet of *S*. *pilchardus*, such as crustacean eggs, copepods, decapods, cirripedes, dinoflagellates and diatoms (Garrido et al., 2008). The lower trophic position of this fish species in the trophic web may reflect a reduced likelihood of contact with infective L3 larvae. However, the limited sample size in the present study (*n* = 20) could affect the representativeness of the reported prevalence and therefore its interpretation. In contrast, the remaining analysed hosts are mostly carnivores and feed on other transport host fishes (Cabral and Murta, 2002), which may explain the higher infection levels. These results collectively reinforce the strong influence of host feeding ecology on infection parameters (Mattiucci et al., 2018).

Overall, *A*. *pegreffii* was the predominant species molecularly identified in this study, representing approximately 56.6% of the analysed larvae, followed by *A. simplex* (*s.s.*) (30.9%), and putative hybrid genotypes of the two species (12.3%). In *Scomber colias*, 19.6% of the larvae analysed were molecularly identified as *A. simplex* (*s.s.*) and 71.6% as *A. pegreffii*, while in *S. scombrus* these two parasite species made up 50.8% and 39.3%, respectively. These results are consistent with previous data reported from Portuguese waters, where *A. simplex* (*s.s.*) represented 18% and *A. pegreffii* represented 82% of the analysed larvae collected from *S. colias*; and 73% and 27%, respectively, from *S. scombrus* (Santos et al., 2017). However, it is relevant to notice that in the report by Santos et al. (2017), the capture localities of the hosts were not specified in detail (FAO area Atlantic Northeast 27, subarea Portuguese waters IX), while in the present survey, *S. colias* specimens were sampled off the centre and south of the country and those of *S. scombrus* were mostly collected off northern localities. A similar pattern was observed in *M*. *merluccius* (only sampled off Viana do Castelo, the northernmost locality in the present study), which exhibited a predominance of larvae identified as *A. simplex* (*s.s.*) (68.4%) in contrast to *A. pegreffii*, which only made up 15.8% of the sampled worms. Previously, another study reported that the larvae from the same host collected off a nearby locality (Matosinhos) comprised 70% *A. simplex* (*s.s.*) and 30% *A. pegreffii* (Santos et al., 2022).

As far as *T*. *trachurus* is concerned, presently collected from all sampled localities, most larvae were identified as *A. pegreffii*, representing 57.9% of the overall larvae collected from this fish species, in contrast with *A. simplex* (*s.s.*), which accounted for 27.9%. These findings are aligned with the results of Lopes et al. (2020), who reported 37.1% and 62.9% of the analysed larvae identified as *A. simplex* (*s.s.*) and *A. pegreffii*, respectively, in the same host (Lopes et al., 2020).

In fact, the distribution of anisakid species appeared to follow a geographical and seasonal pattern rather than a host-related one. Larvae of *A. simplex* (*s.s.*) dominated in the northern localities (off Viana do Castelo, Matosinhos, and Figueira da Foz), particularly during the winter, whereas those of *A. pegreffii* were more frequent in the southern areas (off Peniche, Sesimbra, and Portimão). These differences in the relative proportions of *A. simplex* (*s.s.*) and *A. pegreffii* larvae were statistically significant, not only spatially, where the former was overall more frequently detected in the northern localities (*ꭓ*^2^ = 16.6 for *T. trachurus*, *P* < 0.001 and GLM overall, *ꭓ*^2^ = 17.95, *df* = 5, *P* = 0.003), but also seasonally, in which *A. simplex* (*s.s.*) was also more prevalent during winter (*ꭓ*^2^ = 9.72 for *T. trachurus*, *P* = 0.002 and GLM overall, ꭓ^2^ = 4.224, *df* = 1, *P* = 0.040). These patterns seem to confirm the north-south gradient of the two sibling species previously suggested in European waters, with L3 larvae of *A. simplex* (*s.s.*) generally associated with paratenic hosts of the oceanic waters of the Atlantic and those of *A. pegreffii* with fishes from the Mediterranean Sea (Mattiucci et al., 2004, 2018; Mattiucci and Nascetti, 2008).

Interestingly, the present results also appear to underline the plasticity of the distribution of the two parasite species regarding the time of year. These findings support the hypothesis that the distinct segregation of these sibling species, among other factors, may be influenced by the temperature of water as previously pointed, where the earlier life stages of *A. simplex* (*s.s.*) appear more adapted to colder waters, while those of *A. pegreffii* demonstrate more tolerance to warmer environments (Gomes et al., 2023a, 2023b). The seasonal variation in water temperatures across the Portuguese coast is well documented. Data from Copernicus Marine Service showed warmer sea surface temperatures along the southern coast (approximately 18 °C in February and 22 °C in July) than in the north (15 °C and 18 °C, during the same months, respectively) of Portugal, in 2024 (Copernicus Marine Service, 2025).

A relevant limitation to this analysis lies on low and variable number of molecularly identified larvae in some of the pools, which may not accurately represent the true species composition, particularly in highly infected hosts. As a result, less abundant species may be underrepresented, while dominant taxa may be proportionally overrepresented. Consequently, the observed species composition should be interpreted as indicative of general patterns rather than precise quantitative estimates. This limitation may also influence comparisons of species distribution across host species and sites of infection and should be considered when considering potential implications for risk assessment, as rare species may have been overlooked.

Molecular identification also confirmed the presence of putative hybrid genotypes of *A. simplex* (*s.s.*) × *A. pegreffii* across nearly all hosts and localities. The widespread occurrence of these genotypes corroborates previous findings off the Iberian Peninsula and western Mediterranean, a sympatric area between the two taxa (Eissa et al., 2018; Roca-Geronès et al., 2021). Nonetheless, these results should be interpreted with caution, as it has been previously demonstrated that the use of a single genetic marker, such as PCR-RFLP analysis of the ITS rDNA region, is not sufficient to reliably identify true hybrid genotypes (Mattiucci et al., 2016). Consequently, some of the specimens classified as putative hybrids may have been misidentified, reflecting methodological limitations rather than actual hybridization events. In fact, it has been estimated that approximately two-thirds of the number of larvae identified as putative hybrids using PCR-RFLP of rDNA do not exhibit hybrid genotypes using other nuclear diagnostic loci (Mattiucci et al., 2016). In the present study, 52 specimens (out of 424) were identified as putative hybrids and excluded from the analyses. If these larvae had been more accurately identified, the interpretation of species composition and distribution patterns might have been clearer, possibly with larger differences in the numbers of larvae assigned to each sibling species.

The detection of a single larva of *S*. *physeteris* in *T. trachurus* (off Portimão; winter) represents an interesting incidental finding, as this species is typically associated with oceanic fish hosts, such as *Xiphias gladius* (Mattiucci et al., 2014) and sperm whales (*Physeter macrocephalus*) (Blažeković et al., 2015). Its sporadic occurrence in the south Portugal waters may reflect an occasional interaction of coastal fish with deep water cetacean definitive hosts.

Although most larvae were localized in the viscera of the fish, a non-negligible part was detected in the musculature of the analysed specimens. Regarding the distribution of the parasite species, larvae of *A. simplex* (*s.s.*) were sometimes as frequent in the viscera as in the muscles (*S. colias* in winter and *T. trachurus* in summer), occasionally more prevalent in the muscles than in the viscera (*T. trachurus* in winter and *S. colias* in summer) and in some cases more frequent in the viscera than in the muscles (*S. scombrus* in winter and summer) (Fig. 4). On the other hand, larvae of *A. pegreffii* were constantly more frequently detected in the viscera than in the muscles of the analysed fish, except for *S. scombrus* in the winter.

Larval migration from the viscera (initial place of infection) to the muscles of fish may occur *intra vitam* (during the host’s life) or postmortem (Mattiucci et al., 2018). The extent of this migration may depend on several factors, such as host species, storage temperature and time, but also parasite species. The present results should be interpreted cautiously because although fish were always transported on ice and the time between capture (and therefore fish death) and sample processing was approximately 1 day in most localities, occasionally this period reached 3 days (Matosinhos and Viana do Castelo in winter) due to transport-related logistical issues. Furthermore, some samples were also frozen (from Matosinhos and Viana do Castelo), which may have further influenced larval detectability later during tissue digestion. In addition, molecular subsampling could have partly influenced the observed anatomical distribution patterns, particularly in sample pools where the percentage of analysed larvae was relatively low in contrast to the number of available larvae (Table 3, Table 4), such as in larvae from the viscera.

Although some studies have shown that third-stage larvae of *A. pegreffii* usually exhibit higher penetration ability in agar models (Jeon and Kim, 2015; Kojima and Fujimoto, 2019; Gomes et al., 2023a); other reports have demonstrated that larvae of *A. simplex* (*s.s.*) show greater migratory activity *in vitro* and *in vivo* (Suzuki et al., 2010; Quiazon et al., 2011; Arizono et al., 2012). Migratory activity of larvae of the genus *Anisakis* is a highly relevant issue for food safety, as both *A. simplex* (*s.s*.) and *A. pegreffii* are zoonotic and were detected in the musculature (the primary edible tissue) of these popular fishery products (EFSA Panel on Biological Hazards (BIOHAZ), 2010;EFSA Panel on Biological Hazards (BIOHAZ), 2024). This is especially relevant in Portugal, where fish is traditionally bought and consumed ungutted.

These findings underscore the critical importance of implementing multiple control measures to reduce the risk of anisakid presence in fishery products. Key strategies include the immediate evisceration of fish and storage at temperatures close to that of melting ice immediately after capture, which may reduce postmortem migration of larvae into muscle tissue. For fish intended for raw or lightly processed consumption, strict adherence to freezing protocols as mandated by EU Regulations No. 853/2004 and No. 1276/2011 (European Commission, 2004, 2011) is essential (e.g. −20 °C for at least 24 h, noting that longer periods may be required when using standard domestic freezers). Furthermore, a conscientious selection of fish species used to prepare these raw specialties is advised to reduce the risk of parasite larvae in the muscles. For instance, the present results suggest that *S*. *pilchardus* and *S*. *scombrus*, particularly smaller specimens, exhibit both a lower probability of presence of anisakid larvae and a lower estimated number of larvae per infected host in the muscles (Fig. 2, Fig. 3). Additionally, thorough cooking of fish at temperatures exceeding 60 °C for at least 1 min effectively inactivates larvae preventing infective anisakiasis, while careful visual inspection of fishery products remains a practical supplementary measure to detect and remove visible parasites. Nevertheless, prevention of allergic reactions in sensitized consumers is still a challenge because allergens of *Anisakis* spp. larvae are highly temperature and pepsin-resistant (Rama and Silva, 2022).

While several methodological aspects have been addressed throughout this section, it is important to explicitly acknowledge the main limitations of the present study. These include potential biases related to sample processing (e.g. prior evisceration and freezing), the relatively limited number of larvae subjected to molecular identification, and constraints associated with the use of a single genetic marker, which may have affected species assignment. In addition, the uneven geographical and seasonal sampling design may limit the generalization of the observed patterns. Collectively, these factors should be considered when interpreting the results, particularly regarding species composition, infection levels, and their spatial and temporal variability.

Despite these limitations, the present study provides robust and relevant insights into the occurrence and distribution of *Anisakis* spp. in common fishes from Portuguese waters. *Anisakis pegreffii* was identified as the predominant species, while *A. simplex* (*s.s.*) also showed a relevant presence, particularly in northern localities and during winter, with a notable occurrence in fish muscles, suggesting both a seasonal and geographical variation. Although most larvae were confined to the viscera, their detection in edible tissues highlights the importance of continuous monitoring and the implementation of strict post-capture handling measures. Overall, these findings contribute to a better understanding of the ecological dynamics and epidemiological relevance of *Anisakis* spp., reinforcing their significance for both fisheries management and consumer safety.

## 5 Conclusions

This study provides a comprehensive and updated assessment of *Anisakis* spp. in commercially valuable fish species off the Portuguese coast, revealing a highly dynamic and ecologically complex reality. Our findings confirm a clear geographical and seasonal segregation between the two main zoonotic sibling species, with *A*. *simplex* (*s.s.*) dominating northern waters during winter and *A*. *pegreffii* exhibiting higher prevalence in southern regions, particularly in the viscera of fishes. Despite inherent sampling and methodological limitations, these results strongly support the hypothesis that ambient water temperature may influence anisakid infection dynamics in the waters of the Atlantic-Mediterranean transition. Crucially, the non-negligible presence of both species as well as their putative hybrids in the muscle tissue of fish hosts highlights a persistent risk for consumers. Ultimately, this work underscores the critical importance of strict adherence to food safety regulations as well as sustained epidemiological surveillance in safeguarding public health and minimizing the economic impacts of the presence of anisakid larvae in fishery products.

## Ethical approval

This study received approval from the Ethics and Animal Welfare Commission of the Faculty of Veterinary Medicine of Lusófona University (no. 32-2023).

## CRediT authorship contribution statement

**Beatriz Mendes:** Formal analysis, Investigation, Visualization, Writing – original draft. **Duarte Marques:** Investigation. **Monalisa Medeiros:** Investigation. **Leonor Abegão:** Investigation. **Inês Mira:** Resources, Writing – review & editing. **Ana Maria Munhoz:** Conceptualization, Funding acquisition, Investigation, Resources, Supervision, Visualization. **Sónia Ramos:** Conceptualization, Funding acquisition, Investigation, Resources, Supervision, Writing – original draft, Writing – review & editing. **Tiago Leandro Gomes:** Conceptualization, Data curation, Formal analysis, Funding acquisition, Investigation, Methodology, Project administration, Resources, Supervision, Validation, Visualization, Writing – original draft, Writing – review & editing.

## Funding

This study was part of project FishParaZ (SUB003118) funded by I-MVET through ILIND – Lusófona University.

## Declaration of competing interests

The authors declare that they have no competing financial interests or personal relationships that could have appeared to influence the work reported in this paper.

## Data Availability

The data supporting the conclusions of this article are included within the article and its supplementary file. The newly generated ITS sequences of *A. simplex* (*s.s.*), *A. pegreffii* and *S. physeteris* were deposited in the GenBank database under the accession numbers PX939965-PX939966, PX939976, PX940053, PX940055-PX940056, PX940097, PX940532 and PX940547.

## Appendix A Supplementary data

Supplementary file 1 Supplementary Table S1**.** Generalized Linear Model (GLM) outputs for the predictors of parasite prevalence in the viscera of sampled fish hosts. Supplementary Table S2. Generalized Linear Model (GLM) outputs for the predictors of parasite prevalence in the muscles of sampled fish hosts. Supplementary Table S3. Generalized Linear Model (GLM) outputs for the predictors of parasite intensity in the viscera of sampled fish hosts. Supplementary Table S4. Generalized Linear Model (GLM) outputs for the predictors of parasite intensity in the muscles of sampled fish hosts. Supplementary Table S5. Comparison of sequence identity percentages for the ITS regions (ITS1-5.8S-ITS2) between anisakid samples. Supplementary Table S6. Binary Logistic Regression model (GLM) outputs for the predictors of parasite species.
